# Supplementary material for: Evaluation of Non-Vector Transmission of Usutu Virus in Domestic Canaries (Serinus canaria)
Source: Viruses. 2024 Jan 3;16(1):79. doi: 10.3390/v16010079 (PMC10819963; doi:10.3390/v16010079)
Supplement: Supplementary file 1 [file viruses-16-00079-s001.zip › viruses-2739652-supplementary.pdf]

Canary	Inoculation route	Mortality	Swab Ct			Infectious virus isolation		
			2 dpi	4 dpi	6 dpi	2 dpi	4 dpi	6 dpi
**A1**	**10^6 TCID$_{50}$ i.d.**	1	31.04	25.57	23.22	-	+	+
**A2**		0	37.01	29.89	37.01	-	+	-
**A3**		1	24.75	23.21	24.37	+	+	+
**A4**		0	34.06	22.45	30.71	-	+	-
**A5**		0	33.03	25.46	30.01	-	+	-
**A6**		0	35.02	24.33	34.61	-	+	-
**B1**	**i.d. sentinels**	0	UND.	UND.	UND.	-	-	-
**B2**		0	UND.	37.77	37.92	-	-	-
**B3**		0	UND.	37.88	36.95	-	-	-
**B4**		0	UND.	UND.	38.00	-	-	-
**B5**		0	UND.	UND.	38.07	-	-	-
**B6**		0	UND.	38.15	37.85	-	-	-
**C1**	**10^6 TCID$_{50}$ i.n.**	0	36.98	37.59	37.23	-	-	-
**C2**		0	36.75	37.62	36.33	-	-	-
**C3**		0	30.17	24.75	22.86	-	+	+
**C4**		0	29.01	22.46	28.13	+	+	+
**C5**		0	37.76	37.62	35.67	-	-	-
**C6**		1	26.85	24.17	22.58	+	+	+
**D1**	**10^4 TCID$_{50}$ i.n.**	0	UND.	37.96	37.58	-	-	-
**D2**		0	UND.	37.92	37.62	-	-	-
**D3**		1	31.66	25.23	26.82	-	+	+
**D4**		0	UND.	37.83	UND.	-	-	-
**D5**		0	UND.	37.98	UND.	-	-	-
**D6**		0	UND.	35.73	37.75	-	-	-
**E1**	**10^2 TCID$_{50}$ i.n.**	0	UND.	36.96	38.74	-	-	-
**E2**		0	36.60	37.94	UND.	-	-	-
**E3**		0	UND.	38.02	35.70	-	-	-
**E4**		0	UND.	37.89	UND.	-	-	-
**E5**		0	UND.	UND.	UND.	-	-	-
**E6**		0	UND.	UND.	UND.	-	-	-
**F1**	**i.n. sentinels**	0	UND.	UND.	35.92	-	-	-
**F2**		0	UND.	UND.	36.63	-	-	-
**F3**		0	UND.	UND.	35.39	-	-	-
**F4**		0	UND.	UND.	35.00	-	-	-
**F5**		0	UND.	UND.	37.68	-	-	-
**F6**		0	UND.	UND.	36.05	-	-	-
